# Supplementary material for: Cocoa Nanoparticles to Improve the Physicochemical and Functional Properties of Whey Protein-Based Films to Extend the Shelf Life of Muffins
Source: Foods. 2021 Nov 3;10(11):2672. doi: 10.3390/foods10112672 (PMC8622579; doi:10.3390/foods10112672)

Figure. S1. Appearance of the (**A**) protein solution with 8% WPC and 6% glycerol (G); and (**B**) nanoemulsion with 8% WPC, 6% G and 2% CL.

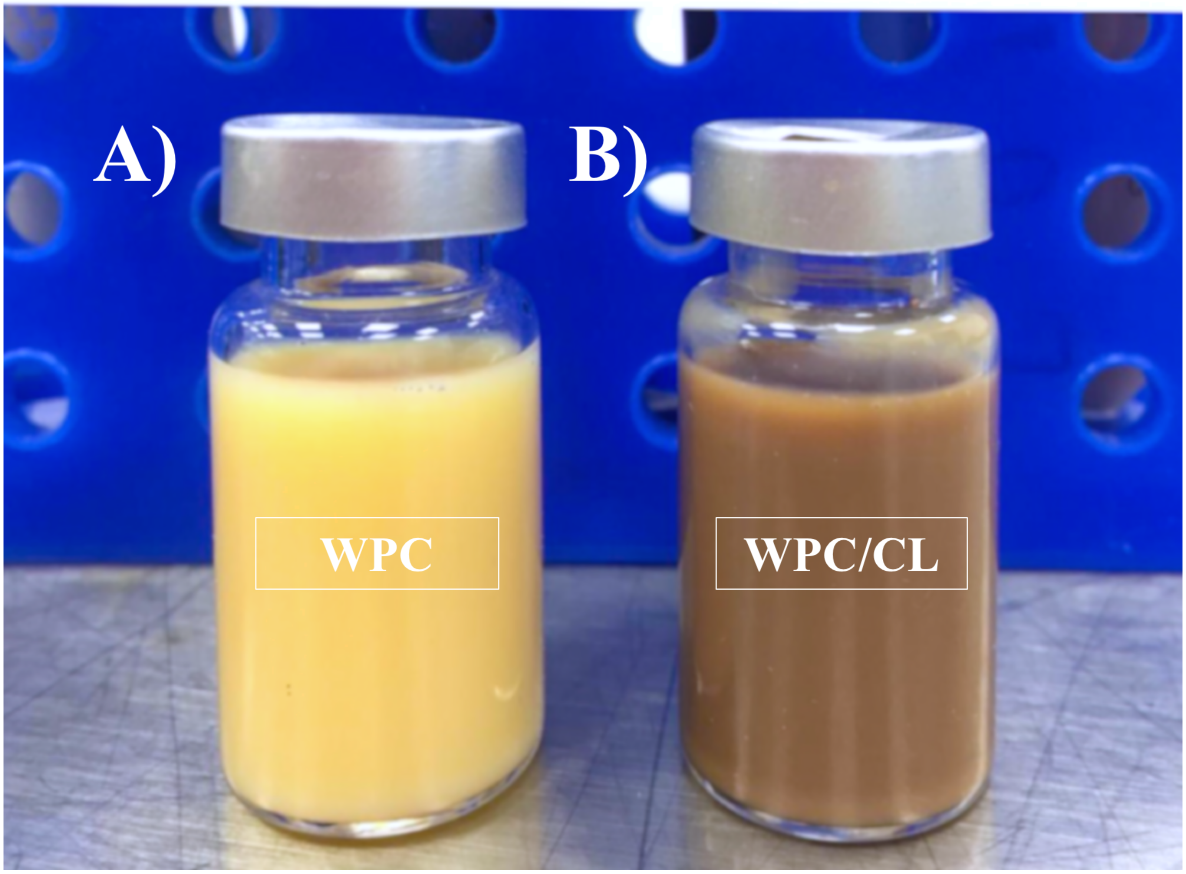

Figure. S2. Appearance of the film obtained from (**A**) protein solution with 8% WPC and 6% glycerol (G); and (**B**) nanoemulsion with 8% WPC, 6% G and 2% CL.

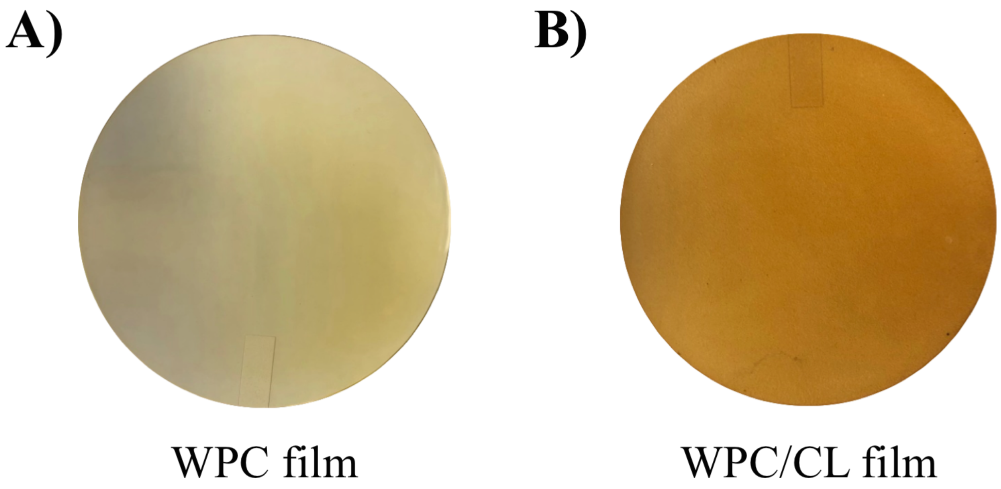

Figure. S3. Appearance of the muffins (**A**) uncoated (Control); (**B**) Coating WPC (with coating 8% WPC and 6% glycerol; and (**C**) Coating WPC/CL (with coating 8% WPC, 6% glycerol, and 2% cocoa liquor).

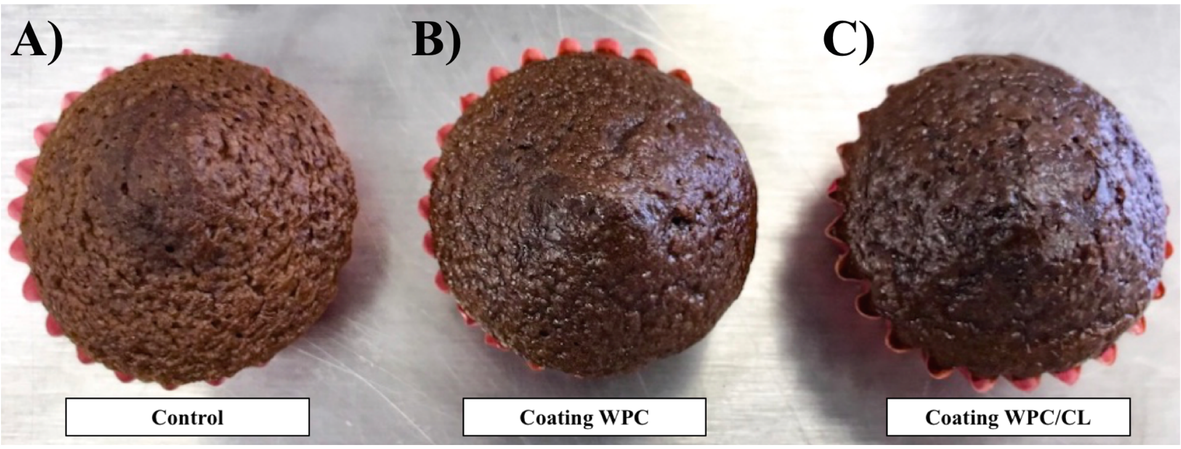

Supplement: Supplementary file 1 [file foods-10-02672-s001.zip › foods-1386962-supplementary.pdf]
